# Supplementary material for: MetaRibo-Seq measures translation in microbiomes
Source: Nat Commun. 2020 Jun 29;11:3268. doi: 10.1038/s41467-020-17081-z (PMC7324362; doi:10.1038/s41467-020-17081-z)
Supplement: Supplementary file 10 — Supplementary Data 7 [file 41467_2020_17081_MOESM10_ESM.zip › File2/Confidence_High_Taxonomy/409576_out.krona.html]

Javascript must be enabled to view this page.

members
magnitude
magnitudeUnassigned
count
unassigned
taxon
rank

409576\_out

187

superkingdom
187
2

phylum
186
976

class
200643
186

186
171549
order

815
184
family

3

SRS075398\_contig\_number\_contig-100\_2479.23240SRS1041138\_contig\_number\_14714SRS105082\_contig\_number\_3581
genus
184
816

2292944
3
species

SRS1055038\_contig\_number\_1495SRS143991\_contig\_number\_11227SRS148874\_contig\_number\_5840

1262753
1

SRS1041092\_contig\_number\_11204
species

817
1

SRS013098\_contig\_number\_36517
species

1
1638779
species

SRS097920\_contig\_number\_27199


SRS075341\_contig\_number\_12684SRS142712\_contig\_number\_3049SRS149325\_contig\_number\_14183
species
246787
3

species

SRS893358\_contig\_number\_3740
46506
1

1263042
3

SRS050299\_contig\_number\_12375SRS056259\_contig\_number\_contig-100\_1997.159505SRS058070\_contig\_number\_28303
species


SRS015190\_contig\_number\_10613SRS043411\_contig\_number\_11459SRS050801\_contig\_number\_741SRS051031\_contig\_number\_21645
species
4
820

357276
18
species

SRS013940\_contig\_number\_19436SRS016989\_contig\_number\_2349SRS017859\_contig\_number\_contig-100\_390.56001SRS018541\_contig\_number\_7319SRS018575\_contig\_number\_8151SRS045195\_contig\_number\_6334SRS048981\_contig\_number\_1868SRS052027\_contig\_number\_2615SRS056519\_contig\_number\_4147SRS075021\_contig\_number\_2697SRS077024\_contig\_number\_2965SRS077231\_contig\_number\_14454SRS098717\_contig\_number\_2504SRS1041112\_contig\_number\_57SRS1041112\_contig\_number\_5636SRS1055034\_contig\_number\_9313SRS149784\_contig\_number\_22779SRS149879\_contig\_number\_26905

species

SRS144753\_contig\_number\_12112
2292943
1


SRS012273\_contig\_number\_37302SRS058770\_contig\_number\_3916SRS077392\_contig\_number\_31610
species
28116
3

671267
2

SRS1041129\_contig\_number\_13993SRS143876\_contig\_number\_13887
species

species

SRS015890\_contig\_number\_4625SRS018984\_contig\_number\_3769SRS020233\_contig\_number\_42279SRS022071\_contig\_number\_contig-100\_1528.221889SRS893230\_contig\_number\_8789
457395
5

28111
1

SRS050752\_contig\_number\_2491
species

821
134
species

SRS011302\_contig\_number\_21754SRS012849\_contig\_number\_contig-100\_2751.178414SRS012902\_contig\_number\_677SRS013638\_contig\_number\_4994SRS013965\_contig\_number\_38691SRS014459\_contig\_number\_41435SRS014683\_contig\_number\_7020SRS014855\_contig\_number\_7397SRS015065\_contig\_number\_38788SRS015431\_contig\_number\_70265SRS015663\_contig\_number\_15649SRS015694\_contig\_number\_17202SRS015782\_contig\_number\_47164SRS015854\_contig\_number\_12489SRS015890\_contig\_number\_6975SRS015960\_contig\_number\_4979SRS016056\_contig\_number\_3118SRS016132\_contig\_number\_2204SRS016203\_contig\_number\_1547SRS016840\_contig\_number\_2248SRS016954\_contig\_number\_25025SRS017375\_contig\_number\_533SRS017701\_contig\_number\_contig-100\_2097.120610SRS018427\_contig\_number\_3873SRS018817\_contig\_number\_10891SRS018836\_contig\_number\_19920SRS018888\_contig\_number\_9789SRS019030\_contig\_number\_14007SRS019397\_contig\_number\_11515SRS020508\_contig\_number\_341SRS020622\_contig\_number\_3109SRS020869\_contig\_number\_29953SRS021153\_contig\_number\_contig-100\_149.35164SRS021948\_contig\_number\_23892SRS023829\_contig\_number\_421SRS024009\_contig\_number\_contig-100\_766.72887SRS042284\_contig\_number\_904SRS044535\_contig\_number\_11453SRS045826\_contig\_number\_7467SRS046502\_contig\_number\_13716SRS046810\_contig\_number\_contig-100\_32.33SRS048164\_contig\_number\_13274SRS048164\_contig\_number\_31625SRS049446\_contig\_number\_9649SRS049712\_contig\_number\_24153SRS049773\_contig\_number\_6090SRS049959\_contig\_number\_36264SRS050941\_contig\_number\_15518SRS052576\_contig\_number\_90SRS053087\_contig\_number\_contig-100\_121.37948SRS054352\_contig\_number\_12744SRS054590\_contig\_number\_143SRS054905\_contig\_number\_2093SRS055017\_contig\_number\_34654SRS056273\_contig\_number\_4968SRS057480\_contig\_number\_contig-100\_34.68136SRS058268\_contig\_number\_66SRS062654\_contig\_number\_21954SRS062701\_contig\_number\_1777SRS063518\_contig\_number\_15548SRS063928\_contig\_number\_contig-100\_765.766SRS074586\_contig\_number\_4925SRS075078\_contig\_number\_11741SRS075398\_contig\_number\_126SRS075773\_contig\_number\_contig-100\_1750.231635SRS075821\_contig\_number\_15911SRS075984\_contig\_number\_15796SRS076756\_contig\_number\_contig-100\_1062.123569SRS076876\_contig\_number\_176SRS076976\_contig\_number\_16102SRS077127\_contig\_number\_contig-100\_2205.110386SRS077294\_contig\_number\_contig-100\_1422.109969SRS077454\_contig\_number\_14882SRS077552\_contig\_number\_13633SRS077991\_contig\_number\_contig-100\_68.23975SRS078242\_contig\_number\_20969SRS097889\_contig\_number\_14792SRS098073\_contig\_number\_13554SRS098571\_contig\_number\_20385SRS098644\_contig\_number\_19183SRS098827\_contig\_number\_2330SRS098881\_contig\_number\_1121SRS103987\_contig\_number\_15412SRS104036\_contig\_number\_7104SRS1041031\_contig\_number\_29301SRS1041036\_contig\_number\_1352SRS1041090\_contig\_number\_contig-100\_141.50208SRS1041091\_contig\_number\_18127SRS1041116\_contig\_number\_9152SRS1041133\_contig\_number\_19924SRS1041134\_contig\_number\_11426SRS1041142\_contig\_number\_1683SRS1041144\_contig\_number\_4705SRS1041157\_contig\_number\_5193SRS1041164\_contig\_number\_2695SRS104327\_contig\_number\_3127SRS104400\_contig\_number\_25128SRS1054928\_contig\_number\_2478SRS1055022\_contig\_number\_12527SRS1055049\_contig\_number\_contig-100\_1563.81853SRS1055056\_contig\_number\_12337SRS1055083\_contig\_number\_4556SRS142542\_contig\_number\_23980SRS142923\_contig\_number\_20044SRS143070\_contig\_number\_4283SRS143148\_contig\_number\_25499SRS143181\_contig\_number\_14327SRS143417\_contig\_number\_32903SRS143523\_contig\_number\_21028SRS143722\_contig\_number\_4021SRS143780\_contig\_number\_1456SRS143895\_contig\_number\_31802SRS144537\_contig\_number\_51292SRS144603\_contig\_number\_12327SRS145308\_contig\_number\_787SRS145497\_contig\_number\_15880SRS146764\_contig\_number\_5065SRS147022\_contig\_number\_22SRS147039\_contig\_number\_1822SRS147614\_contig\_number\_5183SRS148196\_contig\_number\_34092SRS148253\_contig\_number\_3660SRS148424\_contig\_number\_contig-100\_30.174800SRS148817\_contig\_number\_9730SRS149244\_contig\_number\_9705SRS893170\_contig\_number\_4198SRS893253\_contig\_number\_1186SRS893279\_contig\_number\_12168SRS893341\_contig\_number\_14242SRS893366\_contig\_number\_19829SRS893369\_contig\_number\_8115SRS893378\_contig\_number\_13340SRS893383\_contig\_number\_26523SRS971276\_contig\_number\_28752

2
1853231
family

genus
1
574697

1472416
1
species

SRS016018\_contig\_number\_11342

283168
1
genus

species

SRS077194\_contig\_number\_13202
28118
1

1
1239
phylum

class
186801
1

186802
1
order

1
186803
family


SRS148784\_contig\_number\_18043
species
1898203
1
